# Trade-offs in quality of life and survival with chemotherapy for advanced breast cancer: mature results of a randomized trial comparing single-agent mitoxantrone with combination cyclophosphamide, methotrexate, 5-fluorouracil and prednisone

**DOI:** 10.1186/2193-1801-2-391

**Published:** 2013-08-21

**Authors:** Chee Khoon Lee, Val J Gebski, Alan S Coates, Anne-Sophie Veillard, Vernon Harvey, Martin HN Tattersall, Michael J Byrne, Brian Brigham, John Forbes, R John Simes, on behalf of Australia and New Zealand Breast Cancer Trials Group (ANZBCTG)

**Affiliations:** National Health and Medical Research Council Clinical Trials Centre, University of Sydney, Sydney, Australia; International Breast Cancer Study Group, Bern, Switzerland; Regional Cancer and Blood Centre, Auckland City Hospital, Auckland, New Zealand; The University of Sydney, Sydney, Australia; Department of Oncology, Sir Charles Gairdner Hospital, Perth, Australia; Australian New Zealand Breast Cancer Trials Group, University of Newcastle, Newcastle, Australia; ANZ BCTG Statistical Centre, NHMRC Clinical Trials Centre, Sydney, Australia

**Keywords:** Breast cancer, Chemotherapy, Quality of life, Toxicity, Survival, Randomized trial

## Abstract

**Background:**

We evaluate trade-offs between quality of life (QoL) and survival improvement for two chemotherapy regimens in advanced breast cancer. We also report on the long-term survival of patients in the ANZ 8614 clinical trial.

**Methods:**

A total of 391 patients were randomized to mitoxantrone (14 mg/m^2^ intravenously every 21 days) or a combination of cyclophosphamide 100 mg/m^2^ and prednisone 40 mg/m^2^ orally days 1 to 14 plus methotrexate 40 mg/m^2^ and 5-fluorouracil 600 mg/m^2^ intravenously days 1 and 8 every 28 days (CMFP). QoL was self-assessed on 14 linear analog scales. We computed the mean differences between the two treatments as products of the mean differences in global QoL, progression-free survival and overall survival.

**Results:**

CMFP led to a higher overall tumor response (39% vs. 25%, *P*=0.004) and longer progression-free survival (PFS) (median 5.6 vs 3.9 months, *P*=0.02) but with significantly more toxicity from alopecia, mucositis, diarrhea, anemia and lethargy. Overall survival (OS) was similar in the two groups (median 10.1 vs 11.6 months, *P*=0.81). QoL over the first 12 weeks was rated better by patients on CMFP for mood (*P*=0.04), nausea and vomiting (*P*=0.01), and feeling sick (*P*=0.02) but worse for hair loss (*P*<0.0001). A weighted combination of individual QoL items favoured CMFP (subset score mean difference 2.4, *P*=0.03). A global QoL score tended to favour CMFP (global score mean difference 1.7, *P*=0.18). Quality-adjusted PFS was significantly longer with CMFP (mean 7.208 vs 5.965 months, *P*=0.04), but quality-adjusted OS was not significantly different (mean 11.832 vs 11.315 months, *P*=0.57).

**Conclusion:**

Despite the greater toxicity, the superior antitumor activity of CMFP led to an overall improvement in quality-adjusted PFS. In advanced breast cancer, in clinical decision making about treatment for palliative intent, the principle used to assess trade-offs between antitumor efficacy and toxicity remains relevant and applicable to all modern therapeutic agents.

## Background

Most patients with advanced breast cancer will die as a result of their disease. Systemic treatment is usually administered with palliative intent, with the aims of prolonging overall survival (OS), and improving patient symptoms and quality of life (QoL). With recent new breast cancer therapeutic agents, there have been modest but meaningful improvements in OS (Chia et al. 2007;Gennari et al. 2005;Dafni et al. 2010). However, improvements in symptoms and QoL remain important goals for this incurable disease (Chung and Carlson 2003).

A recent Cochrane review reported that combination chemotherapy resulted in significant prolongation of OS and time to progression and superior tumor response but more toxicity in women with metastatic breast cancer (Carrick et al. 2009). Our previous studies demonstrated that QoL can be improved through chemotherapy despite its treatment-related toxicity (Coates et al. 1987;Stockler et al. 2011). It has been suggested that further improvement in patient outcomes could be achieved by using treatments of low toxicity with the focus on trade-off between QoL and OS (Tannock 1987). However, the interpretation of such a clinical trial could be complicated, particularly when a treatment improves one clinical endpoint, such as QoL, but disadvantages the other, such as OS.

Using data from ANZ 8614 clinical trial (Simes et al. 1994;Bishop et al. 1999;Carrick et al. 2009;Lord et al. 2008), we evaluate trade-offs between QoL and survival improvement, in terms of quality-adjusted survival outcomes, by comparing patients with advanced breast cancer treated with single-agent mitoxantrone and combination cyclophosphamide, methotrexate, 5-flurouracil, and prednisone (CMFP). Mitoxantrone has some clinical activity in advanced breast cancer, is easy to administer and is relatively low in toxicity (Cornbleet et al. 1984). CMFP, on the other hand, is a more complex regimen with established activity and possible greater toxicity than mitoxantrone. In this report, we also provide updated long-term survival outcomes of the ANZ 8614 clinical trial (Simes et al. 1994;Bishop et al. 1999;Carrick et al. 2009;Lord et al. 2008).

## Methods

### Patients

Key eligibility criteria included histologically confirmed breast carcinoma, with recurrent or metastatic measurable or evaluable disease, adequate bone marrow reserves (white blood cell count of 4.0 × 10^9^/L or greater and platelet count of 100 × 10^9^/L or greater), adequate hepatic (serum bilirubin less than 20 mol/L) and renal function (creatinine less than 0.15 mmol/L), and an Eastern Cooperative Oncology Group (ECOG) performance status of 0 to 3. Key exclusion criteria were prior cytotoxic chemotherapy for recurrent or metastatic breast cancer, extensive radiotherapy, past history of other cancer, diabetes mellitus, congestive cardiac failure, and symptomatic coronary artery disease. All patients provided signed written informed consent.

Random assignment was performed centrally and balanced dynamically for the treating institution, performance status (ECOG PS 0 to 1 vs 2 to 3), and the presence of liver or brain metastases. Patients were randomly assigned to receive either mitoxantrone or CMFP. On treatment failure, crossover to the other treatment regimen was allowed as second-line therapy for all patients.

### Treatments

Mitoxantrone was given intravenously in a dose of 14 mg/m^2^, repeated every 21 days. CMFP consisted of oral cyclophosphamide 100 mg/m^2^ and oral prednisone 40 mg/m^2^ daily on days 1 through 14, with methotrexate 40 mg/m^2^ and 5-fluorouracil 600 mg/m^2^ administered intravenously on days 1 and 8 and repeated every 4 weeks.

Doses were based on surface area calculated from the lesser of ideal or actual body weight. Treatment was delayed until toxicities other than alopecia had resolved to grade 1 or lower. Subsequent doses of chemotherapy were routinely adjusted on the basis of pretreatment total white cell count (or, when available, on neutrophil counts at the nadir of each cycle) and platelet count.

The initial chemotherapy regimen was continued until disease progression, patient intolerance, or unacceptable toxicity. A maximum cumulative dose of 140 mg/m^2^ of mitoxantrone or a maximum 12 months of CMFP chemotherapy were recommended. However, beyond these times, further treatment was allowed at the discretion of the responsible clinician, with appropriate monitoring.

### Assessments

Patients were assessed before each cycle of chemotherapy: every 3 weeks for those receiving mitoxantrone and 4 weeks for CMFP. Adverse events were rated with the World Health Organization (WHO) criteria (Miller et al. 1981) after each cycle of chemotherapy. Computerized axial tomographic scans of the chest and abdomen and skeletal radionucleotide scans were performed at baseline and every 12 weeks until disease progression, or at an earlier time if disease progression was suspected.

### Study design

The primary aim of this study was to determine whether a chemotherapeutic regimen of established efficacy but more toxicity is associated with an overall net clinical benefit measured in terms of quality-adjusted progression-free survival (PFS) and quality-adjusted OS. The aim of the ANZ 8614 trial was to compare the two randomized regimens in terms of PFS (defined as time from randomization to progression or death), OS (defined as time from randomization to death), objective tumor response (complete plus partial response), and QoL. The objective tumor response was classified according to the WHO criteria as complete response, partial response, no change, or progressive disease (Miller et al. 1981).

QoL was assessed before random assignment, before each cycle of chemotherapy up to 12 weeks, then every 12 weeks thereafter until death. Patients were asked to rate their QoL averaged over the period since their last assessment by using a series of linear analog self-assessment scales. We used the five linear analog self-assessment (LASA) scales developed by Priestman and Baum (Priestman and Baum 1976): physical wellbeing, mood, pain, nausea and vomiting, and appetite. We also used the general life quality scales (GLQ-8) of Coates and colleagues (Coates et al. 1990): feeling anxious/depressed, feeling sick, numbness, hair loss, tiredness, appetite/taste, sexual interest, the thought of having treatment, and an overall QoL index uniscale. In addition, at each of these times, the clinician completed the Spitzer QL-index (Spitzer et al. 1981).

### Statistical considerations

The planned sample size of 450 patients (225 per arm) accrued over 3 years and observed for an additional 3 years was designed to give 80% power to detect a 35% improvement in 12-month OS from 34% to 45% (hazard ratio (HR), 0.74), comparing the mitoxantrone and CMF groups. The final sample size of 391 patients provided adequate power, owing to longer accrual and follow-up periods.

We compared response and toxicity using the exact tests for these ordered categorical data. We constructed Kaplan-Meier curves for PFS and OS and compared them by using the log-rank test for the analyses of these time-to-event data. The influence of baseline factors on treatment effects was assessed by testing for interactions with treatment in Cox proportional-hazards models for PFS and OS. Multivariable analyses for the baseline factors were also undertaken. All analyses were by intention to treat. All *P* values and 95% confidence intervals (CIs) are two sided.

Scores on each QoL scale were compared for the two treatment groups from randomization until first disease progression. Missing components of QoL data were replaced with the mean values of the treatment group. Limited data on QoL from progression until death were available; this was calculated as the mean of the available QoL scores regardless of treatment group.

A global QoL score, derived using a regression function, was a weighted combination of the measured global QoL scale (GLQ-8) and all individual LASA QoL (except sexual interest) items. A subset QoL score was a weighted combination of only individual LASA QoL (except sexual interest) items. A previous publication outlines the detailed methods of derivation of the global and subset QoL scores (Lumley et al. 2001). A utility score was obtained from power transformation of the global QoL score (Lumley et al. 2001).

Measures of net clinical benefit were demonstrated by incorporating survival and QoL outcomes into single measures as quality-adjusted PFS and quality-adjusted OS (Glasziou et al. 1990). Quality-adjusted PFS for each group was the product of its mean utility score and the area under its time-to-progression curve truncated at 24 months (Glasziou et al. 1990). We calculated the mean utility score from randomization to disease progression using generalized estimating equation models for each treatment group. We used an exchangeable correlation structure and robust standard error estimator to account for correlation among repeated measures of QoL. The CIs and *P* values for differences in quality-adjusted PFS between groups were calculated with bootstrap methods. A similar method was adopted for quality-adjusted OS (where QoL after disease progression was calculated as the average of all available QoL scores) and the area under its time-to-death curve truncated at 30 months.

## Results

**Figure 1:**
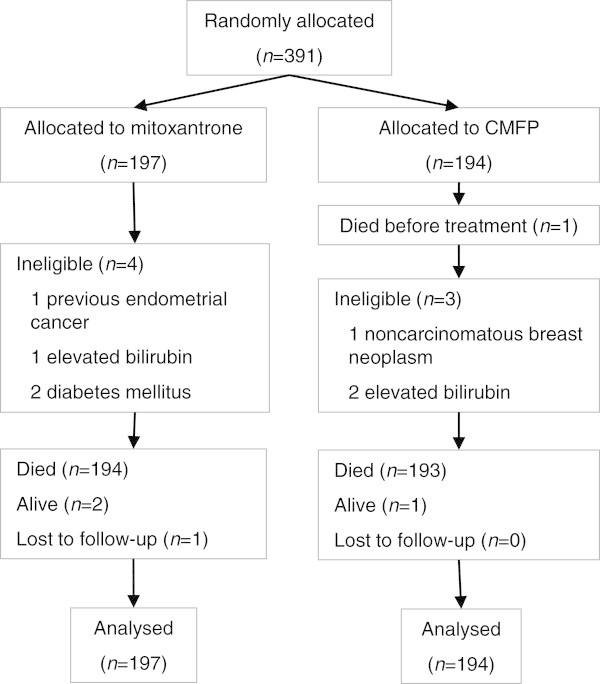
**Enrolment and analysis.**

Between January 1988 and June 1993, 391 patients from 24 institutions in Australia and New Zealand participated in this study (Figure 1). One patient died after randomization but before treatment started. Seven patients were classified as ineligible after randomization. All these eight patients were included in the primary intention-to-treat analysis. All reported analyses are based on 391 patients. Exclusion of the eight ineligible patients did not materially alter the results or conclusions.

At the last data update (June 2008), the median follow-up period was 11.6 years (range, 2 days to 11.9 years), 354 (91%) of the patients had disease progression, 37 (9%) were known to be progression-free, 387 (99%) had died, one was lost to follow-up and 3 (1%) were known to be alive.

Baseline characteristics were well balanced among the treatment groups (Table 1). The median age was 57 years; 11% were aged 70 years or older, and 45% were aged 55 years or younger. One patient was male, 161 (41%) had liver and/or brain metastases, 28% had poor performance status (ECOG PS 2 or 3), 19% had received adjuvant chemotherapy, and 169 (45%) had hormone-receptor–positive tumors, of whom 89% had undergone previous endocrine therapy.

**Table 1:** **Baseline characteristics**

	Mitoxantrone		CMFP	
**Characteristics**	***n***	**%**	***n***	**%**
Age, years				
<50	53	27	63	32
50–59	63	32	48	25
60–69	57	29	65	34
≥70	24	12	17	9
Unknown	0	0	1	<1
Liver or brain metastases	84	43	77	40
Interval from diagnosis of breast cancer to diagnosis of advanced disease ≥2 years	90	46	101	52
ECOG performance status				
0	58	29	58	30
1	84	43	82	42
2	38	19	41	21
3	17	9	13	7
Hormone receptor status				
ER or PR positive	92	48	77	41
ER and PR negative	38	20	37	20
ER and PR unknown	62	32	73	39
Previous chemotherapy	37	19	35	18
Previous endocrine therapy	167	85	158	81

*ER* estrogen receptor, *PR* progesterone receptor.

In the mitoxantrone group, patients received 94% of the recommended initial dosage. Both treatment groups had the same mean number of cycles of chemotherapy (5.4). In the CMFP group, patients received 92% of the recommended initial dosage of cyclophosphamide, 94% of methotrexate, 95% of 5-flourouracil and 92% of prednisone.

### Objective tumor response

Of the 365 patients (93%) evaluable for tumor response, confirmed complete or partial responses to initial randomly assigned therapy were documented in 117 (32%). The objective tumor response rate was significantly greater for CMFP than mitoxantrone (39% vs 25%, *P*=0.001) (Table 2).

**Table 2:** **Tumor response to treatment**

	Mitoxantrone (***n***=187)*		CMFP (***n***=178)*		All patients (***n***=365)*	
**Objective response**	***n***	**%**	***n***	**%**	**N**	**%**
Complete response	4	2	9	5	13	3
Partial response	43	22	61	31	104	27
Stable disease	77	39	78	40	155	40
Progressive disease	63	32	30	15	93	24
Unknown response	10	5	16	8	26	7
Response rate (of evaluable)	47	25.1%	70	39.3%	117	32.1%

* The total number of patients evaluable for response was 365 (CMFP 178, mitoxantrone 187).

CMFP, combination therapy with cyclophosphamide, methotrexate, 5-fluorouracil and prednisone.

Two-hundred and eighteen patients subsequently crossed over to receive the other therapy at first disease progression; 115 patients randomized to mitoxantrone went on to receive CMFP, and 103 randomized to CMFP went on to receive mitoxantrone. Patients who received CMFP as second-line therapy had a significantly higher response rate than those crossing over to mitoxantrone (27% vs 11%, *P*=0.006). Over 60% of patients receiving mitoxantrone as second-line therapy had disease progression without a prior objective tumor response or stable disease. The probability of a response on second-line CMFP was independent of a prior response to mitoxantrone.

### Progression-free survival and overall survival

**Figure 2:**
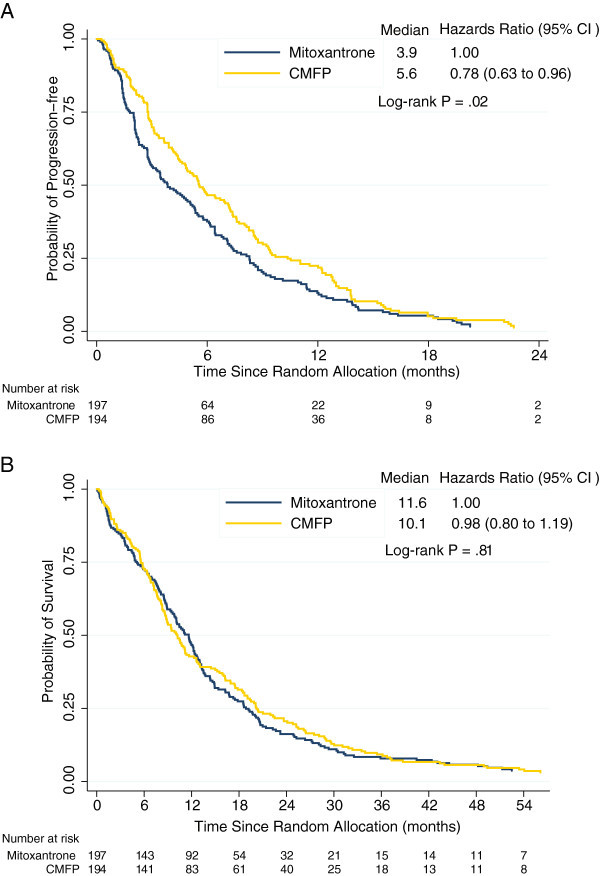
**Progression-free survival (A) and overall survival (B) by treatment group, among women with advanced breast cancer treated with mitoxantrone or CMFP (cyclophosphamide, methotrexate, 5-fluorouracil, prednisone).**

The time to first disease progression was significantly longer for the CMFP than the mitoxantrone group (median PFS 5.6 vs 3.9 months; HR, 0.78; 95% CI 0.63 to 0.96; *P*=0.02, Figure 2A).

OS was similar in the CMFP and the mitoxantrone groups (10.1 vs 11.6 months; HR, 0.98; 95% CI 0.80 to 1.19; *P*=0.81, Figure 2B).

### Adverse events

Adverse events were evaluable in 377 patients (96%) (Table 3). Adverse events of any grade occurred more frequently in patients randomized to CMFP than mitoxantrone, in particular for alopecia, stomatitis, diarrhea, lethargy/somnolence, and anemia. There was no significant difference in leucopenia, thrombocytopenia or nausea/vomiting between the two regimens. When hematologic toxicity was excluded, 52 (27%) of patients receiving CMFP compared with 15 (8%) on mitoxantrone (*P*=0.0001) had at least one episode of toxicity at grade 3 or worse.

**Table 3:** **Adverse events**

	Mitoxantrone (***n***=186)		CMFP (***n***=191)		
	Any grade	Grade 3–4	Any grade	Grade 3–4	
**Adverse event**	***N***	***N***	***n***	***n***	***P*** *
Hematuria	4	0	6	0	0.75
Stomatitis	34	2	91	21	<0.001
Nausea and vomiting	135	10	137	9	0.82
Alopecia	83	0	131	0	<0.001
Diarrhea	19	0	56	2	<0.001
Somnolence	14	0	34	2	0.003
Anemia	83	5	119	15	0.001
Leukopenia	138	56	146	60	0.64
Neutropenia	114	52	109	53	0.29
Thrombocytopenia	23	10	35	15	0.12
Other	68	4	111	30	<0.001

* *P* for any adverse event vs none between the two treatment groups.

CMFP, combination therapy with cyclophosphamide, methotrexate, 5-fluorouracil and prednisone.

There were 15 treatment-related deaths: 11 patients receiving CMFP (infection 7, cardiomyopathy 1, cardiac failure 1, myelosuppression 2) and 4 patients receiving mitoxantrone (infection 2, cardiomyopathy 1, myelosuppression 1).

### Quality of life

Three-hundred and forty-six patients (89%) had a baseline QoL assessment with at least one follow-up assessment in the first 12 weeks after randomization and were included in the main analyses. QoL scores were not available on 25 patients (6%) randomized to CMFP and 20 patients (5%) randomized to mitoxantrone.

Up to 12 weeks after randomization, the average change in the scores of QoL indicators for mood (difference 3.5, *P*=0.02), pain (difference 8.7, *P*<0.01) and feeling anxious/depressed (difference 4.2, *P*<0.01) and thought of treatment (difference 8.7, *P*<0.01) improved significantly from baseline regardless of treatment assignment. Scores for appetite (difference 5.8, *P*<0.01), nausea and vomiting (difference 7.5, *P*<0.01), hair loss (difference 21.9, *P*<0.01), tiredness (difference 8.4, *P*<0.01), and sexual interest (difference 6.9, *P*<0.01), worsened significantly from baseline. Overall QoL, rated either by the patient (GLQ-8 uniscale, *P*=0.26) or clinician (Spitzer QL-Index, *P*=0.44), remained unchanged over this time.

**Figure 3:**
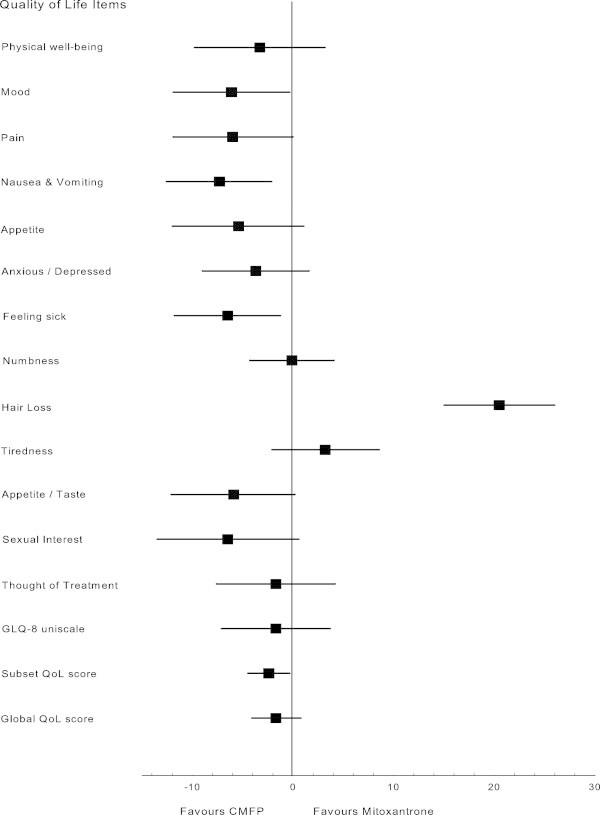
**Difference between quality-of-life scores between patients assigned CMFP and those assigned mitoxantrone, averaged over the first 12 weeks relative to baseline.** The subset QoL score is constructed by using information from all individual LASA QoL (except GLQ-8 and sexual interest) items and is weighted on the basis of patient-rated importance. The global QoL score is constructed by using information from all individual LASA QoL and GLQ-8 (except sexual interest) items and is weighted on the basis of patient-rated importance.

When the average change in QoL in the first 12 weeks after randomization from baseline was compared between the two treatment groups, patients assigned CMFP compared with those assigned mitoxantrone reported better scores for mood (difference 6.1, *P*=0.04), nausea and vomiting (difference 7.3, *P*=0.01), and feeling sick (difference 6.5, *P*=0.02), but worse scores for hair loss (difference 20.5, *P*<0.01) (Figure 3). GLQ-8 uniscale scores rated by the patient and Spitzer QL-Index scores rated by the clinician were not significantly different between treatments (*P*=0.53 and *P*=0.20, respectively). The subset QoL score favored the CMFP group (difference 2.4, *P*=0.03), and the global QoL score had a non-significant trend in favor of CMPF (difference 1.7, *P*=0.18).

When the QoL scores were examined after randomization until first disease progression, similar results were obtained as those up to 12 weeks after randomization (results not shown).

After first disease progression, only 223 patients (57%) had a QoL assessment. QoL scores were not available on 84 patients (43%) randomly assigned mitoxantrone and 84 (43%) randomly assigned CMFP. When QoL was examined over the entire survival period from randomization to death, there were no significant differences, with the exception of hair loss, which was worse for those randomly assigned CMFP (difference 8.7, *P*<0.01).

Quality-adjusted progression-free survival and overall survival analyses are shown in Table 4. Quality-adjusted PFS was significantly longer in patients randomly assigned CMFP than those assigned mitoxantrone (difference 1.243, bootstrapped 95% CI 0.119 to 2.487, *P*=0.04). From first disease progression until death, the average utility for both treatment groups was 0.847. Quality-adjusted OS was similar for patients randomly assigned CMFP and mitoxantrone (Table 4).

**Table 4:** **Quality-adjusted progression-free and Overall Survival**

Parameter	CMFP (***n***=194)	Mitoxantrone (***n***=197)	Difference	95% CI	P*
Mean progression-free months†	7.975	6.666	1.309	0.047 to 2.648	0.05
Mean utility from randomisation to 1st disease progression	0.904	0.895	0.009	−0.006 to 0.022	0.16
Quality-adjusted progression-free survival	7.208	5.965	1.243	0.119 to 2.487	0.04
Mean overall survival months‡	13.330	12.813	0.517	−1.266 to 2.560	0.62
Mean utility from randomisation to death	0.888	0.883	0.005	−0.007 to 0.014	0.35
Quality-adjusted overall survival	11.832	11.315	0.517	−1.120 to 2.296	0.57

* The 95% confidence interval and *P* value were obtained by bootstrap sampling with 1000 replications.

† Mean progression-free survival was calculated as the area under the curve for the time from randomization to first disease progression, truncated at 24 months.

‡ Mean overall survival was calculated as the area under the curve for the time from randomization to death, truncated at 30 months.

CMFP, combination therapy with cyclophosphamide, methotrexate, 5-fluorouracil and prednisone.

## Discussion

Patients randomly assigned CMFP had significantly higher tumor response rate (39% vs 25%, *P*=0.001), significantly longer PFS (median 5.6 vs 3.9 months, *P*=0.02) and significantly longer quality-adjusted PFS (mean 7.208 vs 5.965 months, *P*=0.04) than those assigned mitoxantrone. However, CMFP was significantly associated with at least one episode of grade 3 or worse toxicity than mitoxantrone. Despite the greater toxicity of CMFP, the result of this study was consistent with our hypothesis that better tumor control achieved with this regimen was still associated with improved quality-adjusted PFS for these patients with advanced breast cancer.

Despite a longer PFS with CMFP, there was no advantage with initial use of this regimen in terms of OS or quality-adjusted OS. More than half of the patients whose disease progressed on mitoxantrone were subsequently treated with CMFP. Consequently, post-progression therapy might have obscured any potential advantage of CMFP for OS. In this setting of advanced breast cancer, selection of one of these regimens for initial treatment could reasonably be based on improvement in symptoms and QoL. Despite the additional toxicity of CMFP chemotherapy, patients randomly assigned this treatment reported improvement in QoL after 3 months on therapy (Figure 3), with significant improvement in mood, nausea and vomiting, and feeling sick. There was a nonstatistical significant advantage in favor of CMFP according to the global QoL score, but the quality-adjusted PFS was also significantly longer with CMFP.

Since the time this trial was undertaken, a wide range of newer chemotherapeutic agents and molecularly targeted therapies have become available as first-line treatment for advanced breast cancer. Although neither of the regimens investigated in this randomized trial would now be accepted as the most efficacious first-line therapy, this study has tested an important principle of choosing between the more effective but more toxic CMFP and the less effective and less toxic mitoxantrone. Treatments that are less efficacious but also lower in toxicity may result in fewer treatment-related side-effects, but poor tumor control fails to improve cancer-related symptoms, QoL or quality-adjusted survival outcomes. The results of this study are consistent with our earlier finding (Coates et al. 1987) that optimal patient benefit in advanced breast cancer comes from the use of a regimen likely to be most effective in controlling the tumor. However, when selecting treatment for individual patients, the advantages and disadvantages of each treatment should be considered. For example, mitoxantrone may have a role for those patients who are relatively asymptomatic or who have more slowly evolving forms of cancer and for patients for whom hair loss is a primary concern.

Systematic reviews of chemotherapy in advanced breast cancer have shown survival improvement with combination polychemotherapy (Carrick et al. 2009), and taxane-based chemotherapy (Ghersi et al. 2005), and better tumor response and improved PFS with anthracycline-based chemotherapy (Lord et al. 2008). However, these reviews have reported that gains in survival are modest and these regimens have been associated with additional toxicity. Even with the wide range of modern therapies available, it remains relevant and important that such regimens are assessed for evidence to support a reasonable chance of providing meaningful net clinical benefit through integration of measures of QoL and/or symptom control with survival outcomes as indicators of efficacy in clinical trials.

There are ongoing concerns about the scientific rigor of QoL outcomes and their influence on clinical decision making. A recent review of metastatic breast cancer trials reported that QoL outcomes provided little information beyond that obtained from survival and toxicity outcomes, leading to no change in recommendations as the result of the additional QoL assessments (Goodwin et al. 2003). It has been recommended that QoL assessments would be important when treatments have equivalent tumor-related outcomes and competing toxicities are present or the logistics of treatment administration differ (Goodwin et al. 2003). Our trial results, however, are different, as CMFP is superior to mitoxantrone in tumor-related outcomes, but CMFP is also more toxic than mitoxantrone. In this case, the QoL assessment by patients and the integration of individual QoL scores according to patient weightings with survival outcomes provided a more complete picture of the balance of benefit and harm in guiding clinical decision making. We recommend that this approach be adopted in other clinical trials where trade-offs in toxicity with tumor response and survival exist.

There are several limitations of this study. Both the experimental and control regimens are not the standard choices of first-line therapy in today’s context. However, these choices were reasonable at the time of conception of this trial in the 1980s. The improvement of QoL, such as mood, appetite, nausea, vomiting, could be resulted from the use of prednisone as part of CMFP, but not necessary contributing to the overall efficacy of this regimen. Furthermore, as QoL was assessed subjectively by the patients, some placebo effect may have been involved. However, all patients in this trial received active therapy, and the QoL measures used had been validated, so such an effect, if any, could be considered negligible. Although the global QoL score was calculated by incorporating different QoL items according to the weighted importance from the average trial patient’s perspective, individual patients in other settings may have different trade-offs for these QoL items that may vary from the average perspective. A previous study did demonstrate some similarity in the weights assigned for the QoL items by patients with advanced breast cancer in different settings (Lumley et al. 2001).

## Conclusion

In advanced breast cancer, the main goals of treatment are to improve symptoms and QoL. Many contemporary chemotherapeutic and molecularly targeted agents have different and unique side-effect profiles as compared with the traditional regimens tested in this study. However, the principle used to assess trade-offs between the antitumor efficacy and toxicity remains relevant and applicable for clinical decision making today.
